# Laminin **β**_2_ variants associated with isolated nephropathy that impact matrix regulation

**DOI:** 10.1172/jci.insight.145908

**Published:** 2021-03-22

**Authors:** Yamato Kikkawa, Taeko Hashimoto, Keiichi Takizawa, Seiya Urae, Haruka Masuda, Masumi Matsunuma, Yuji Yamada, Keisuke Hamada, Motoyoshi Nomizu, Helen Liapis, Masataka Hisano, Yuko Akioka, Kenichiro Miura, Motoshi Hattori, Jeffrey H. Miner, Yutaka Harita

**Affiliations:** 1 Department of Clinical Biochemistry, Tokyo University of Pharmacy and Life Sciences, Tokyo, Japan.; 2 Department of Pediatrics, Yamagata University School of Medicine, Yamagata, Japan.; 3 Department of Pediatric Nephrology, School of Medicine, Tokyo Women’s Medical University, Tokyo, Japan.; 4 Department of Pediatrics, Graduate School of Medicine, The University of Tokyo, Tokyo, Japan.; 5 Department of Pathology and Immunology and Department of Medicine, Washington University School of Medicine, St. Louis, Missouri, USA.; 6 Department of Nephrology, Chiba Children’s Hospital, Chiba, Japan.; 7 Department of Pediatrics, Faculty of Medicine, Saitama Medical University, Saitama, Japan.; 8 Division of Nephrology, Department of Medicine, and Department of Cell Biology and Physiology, Washington University School of Medicine, St. Louis, Missouri, USA.

**Keywords:** Cell Biology, Nephrology, Extracellular matrix, Integrins, Laminin

## Abstract

Mutations in *LAMB2*, encoding laminin β_2_, cause Pierson syndrome and occasionally milder nephropathy without extrarenal abnormalities. The most deleterious missense mutations that have been identified affect primarily the N-terminus of laminin β_2_. On the other hand, those associated with isolated nephropathy are distributed across the entire molecule, and variants in the β_2_ LEa-LF-LEb domains are exclusively found in cases with isolated nephropathy. Here we report the clinical features of mild isolated nephropathy associated with 3 *LAMB2* variants in the LEa-LF-LEb domains (p.R469Q, p.G699R, and p.R1078C) and their biochemical characterization. Although Pierson syndrome missense mutations often inhibit laminin β_2_ secretion, the 3 recombinant variants were secreted as efficiently as WT. However, the β_2_ variants lost pH dependency for heparin binding, resulting in aberrant binding under physiologic conditions. This suggests that the binding of laminin β_2_ to negatively charged molecules is involved in glomerular basement membrane (GBM) permselectivity. Moreover, the excessive binding of the β_2_ variants to other laminins appears to lead to their increased deposition in the GBM. Laminin β_2_ also serves as a potentially novel cell-adhesive ligand for integrin α_4_β_1_. Our findings define biochemical functions of laminin β_2_ variants influencing glomerular filtration that may underlie the pathogenesis of isolated nephropathy caused by *LAMB2* abnormalities.

## Graphical abstract

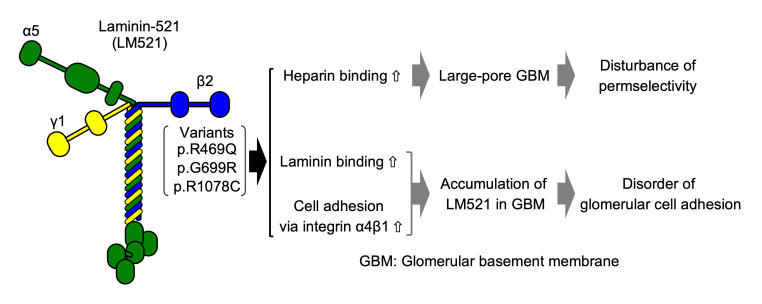

## Introduction

The glomerular filtration barrier allows the efficient flow of water and small molecules while preventing plasma proteins from leaking into the urine. The barrier consists of podocytes, endothelial cells, and the glomerular basement membrane (GBM). Nephrotic syndrome, characterized by proteinuria, edema, hypoalbuminemia, and hyperlipidemia, is associated with impaired glomerular filtration. Nephrotic syndrome can be caused by dysfunctions of podocytes, endothelial cells, or GBM, indicating that all 3 components are essential for maintaining the glomerular filtration barrier. Extensive genetic studies of nephrotic syndrome have identified mutations in genes important for establishing and maintaining the barrier (1).

The GBM is a specialized extracellular matrix separating 2 cellular layers, endothelial cells and podocytes. The GBM plays critical roles in filtration and in maintenance of glomerular morphology (2). Basement membrane (BM) is usually composed of 4 major extracellular matrix proteins: laminin, collagen IV, nidogen, and sulfated proteoglycan. The GBM contains an atypical assortment of BM protein isoforms, including collagen α_3_α_4_α_5_(IV) and laminin-521 (LM521; α_5_β_2_γ_1_; ref. 3). Mutations in collagen α_3_α_4_α_5_(IV) cause Alport syndrome, a kidney disease with variable ear and eye defects (4). LM521 is composed of α_5_, β_2_, and γ_1_ chains. Mutations in the laminin β_2_ gene (*LAMB2*) cause Pierson syndrome, which is characterized by congenital nephrotic syndrome with severe ocular and neuromuscular defects (5). Zenker et al. originally identified homozygous or compound heterozygous mutations in the *LAMB2* gene, which contains 32 exons, in patients with Pierson syndrome (6). More than 49 mutations have since been reported to be associated with Pierson syndrome (5). Most disease-associated alleles are truncating mutations localized across the entire gene, eliminating laminin β_2_ function completely (7). On the other hand, the patients with *LAMB2* missense mutations occasionally exhibit higher mean age at onset of renal disease or oligosymptomatic disease variants of Pierson syndrome (5). For example, p.R246Q and p.R246W lead to congenital or infantile nephrotic syndrome with milder or no extrarenal symptoms (5, 6). A patient with Pierson syndrome carrying p.S80R presented with late-onset nephrotic syndrome that was diagnosed when the patient was 6.5 years old (5). Another missense mutation, p.C321R, is associated with congenital nephrotic syndrome, despite less severe extrarenal defects (5). p.C321R affects the formation of disulfide bonds in an EGF-like module and disrupts the structure of the LEa domain, leading to defective secretion of laminin β_2_ and podocyte ER stress possibly due to misfolding of the aberrant laminin β_2_ (8). Previously, Matejas et al. demonstrated that missense mutations found in Pierson syndrome are apparently clustered in the laminin N-terminal (LN) domain, which is required for the polymerization of laminins (5). Therefore, mutations in the LN domain have been implicated in the perturbation of BM formation.

Laminin is a family of heterotrimeric glycoproteins that mediate cell adhesion via various receptors. Five α, 3 β, and 3 γ chains have been characterized, and 19 isoforms have been identified in various tissues and cell culture media (9). Of the isoforms, 9 contain laminin β_2_, and LM521 is the most studied of these. LM521 trimers are recognized by several receptors, including integrin α_3_β_1_, α_6_β_1_, and α_6_β_4_; α-dystroglycan; and Lutheran/basal cell adhesion molecule. The C-terminal 20–amino acid sequence of the laminin β_2_ chain modulates the binding affinities of laminins to X2-type integrins, such as α_3_β_1_ and α_7_X2β_1_ (10). However, because the laminin globular domains of laminin α chains have been implicated in various cellular interactions, the X2-type integrins are not specific for laminin β_2_. The β_2_ chain of laminin-421 and -521 are also identified as ligands that bind to N- and P/Q-type voltage-gated calcium channels (VGCCs, ref. 11). Although VGCCs specifically bind to laminin β_2_ at neuromuscular junction presynaptic terminals, it is unlikely that these mediate cell adhesion to the β_2_ chain. So far, the cell-adhesive activity of laminin β_2_ has not been fully examined.

Next-generation sequencing approaches have also identified *LAMB2* missense mutations in congenital or childhood-onset nephrotic syndrome without apparent extrarenal abnormalities (12–19). Here we demonstrate that most of these missense variants found in cases with isolated nephropathy affect a region in the LEa-LF-LEb domains in the laminin β_2_ short arm. Notably, variants in this region have not been found in patients with classic Pierson syndrome. We examined the effects of these variants on secretion of laminin β_2_ in vitro. To explore the biochemical properties that might influence glomerular filtration, recombinant laminin β_2_ chains carrying the variants were produced in mammalian cells. We found that laminin β_2_ serves as a potentially novel cell-adhesive ligand for integrin α_4_β_1_. These results unraveled roles of the laminin β_2_ short arm in matrix regulation that may underlie the pathogenesis of isolated nephropathy as a mild form of Pierson syndrome.

## Results

We previously reported a Japanese patient with isolated nephropathy carrying LAMB2 p.R469Q and p.G699R variants (12). He was born to a healthy parent and appeared normal at birth. He exhibited systemic edema by 21 months and was diagnosed with steroid-resistant nephrotic syndrome. A kidney biopsy at 23 months of age revealed focal segmental glomerulosclerosis (FSGS) and mesangial matrix expansion (Supplemental Figure 1A; supplemental material available online with this article; https://doi.org/10.1172/jci.insight.145908DS1). As seen by electron microscopy, the GBM exhibited a stratified structure (Supplemental Figure 1B). The patient’s renal function declined and progressed to end-stage kidney disease (ESKD) at 26 months of age. After renal transplantation at 7 years of age, he is presently healthy without extrarenal symptoms. Our whole-exome analyses of the patient and his parents showed that c.1406G>A (p.R469Q) and c.2095G>C (p.G699R) in *LAMB2* are derived from his father and mother, respectively (Supplemental Figure 2, A and B, and ref. 12). The former, c.1406G>A (p.R469Q), has not been reported in Genome Aggregation Database (gnomAD) (20) or TogoVar (21). Although c.1406G is adjacent to a splice acceptor site, the predictions of bioinformatics tools such as NNSplice and ASSP (22, 23) were comparable to that of WT (Supplemental Figure 2C), suggesting that c.1406G>A does not influence splicing. The allele frequency of c.2095G>C (p.G699R) is 0.01173 in East Asians (gnomAD) and 0.007 in 2 Japanese genome databases (ToMMo 4.7KJPN and GEM-J WGA; available from TogoVar, ref. 21, and Supplemental Table 1).

A Hispanic patient was found to have massive proteinuria by 20 months and diagnosed with steroid-resistant nephrotic syndrome. Sanger sequencing identified a heterozygous c.3232C>T (p.R1078C) variant in *LAMB2*; its allele frequency is 0.000276 (gnomAD). Additional pathogenic variants were not identified in *LAMB2* or in genes associated with early-onset nephrotic syndrome in this patient, but we suspect there is an additional pathogenic variant in *LAMB2* that could not be identified. In silico algorithms (SIFT, PolyPhen-2, Align-GVGD) suggested that p.R1078C is likely to be disruptive (24–26). In the ClinVar database (27) there are multiple submissions of this variant in nephrotic syndrome or Pierson syndrome but with scarce clinical or genetic information.

**Figure 1:**
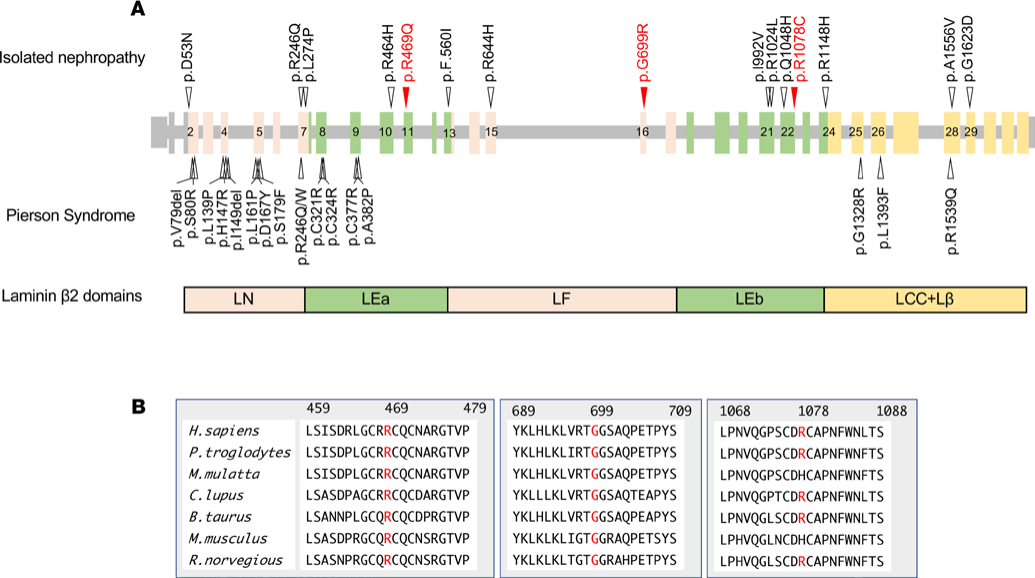
LAMB2 variants. (**A**) The missense variants found in isolated nephropathy (upper arrowheads, Table 1) and Pierson syndrome (lower arrowheads, Supplemental Table 2). Vertical bars and horizontal lines represent exons and introns of the human LAMB2 gene, respectively. Numerals indicate exon numbers. p.R469Q, p.G699R, and p.R1078C are indicated by red arrows. Boxes indicate the domain structure of laminin β_2_. (**B**) Amino acid sequence alignments of laminin β_2_ derived from multiple species.

Including these cases, 14 cases carrying *LAMB2* variants that presented with nephropathy without ocular or neurologic abnormalities have been reported in the literature (Table 1). These patients presented with abnormal urinalysis to varying degrees, ranging from mild hematuria to nephrotic range proteinuria. Among the patients, 4 of their cases progressed to ESKD in the infantile period (cases 2, 3) or in early childhood (cases 4, 5). Other cases maintained normal kidney function in childhood and even at school age. This feature is quite different from the clinical picture of typical Pierson syndrome, in which patients often progress to ESRD in the early infantile period. With respect to genetic involvement, 1 patient (case 14) carries splice site variants in *LAMB2*, and the other patients carry at least 1 missense variant. These missense variants are distributed in exons 2–29 (Figure 1A). This is in clear contrast to missense mutations found in typical patients with Pierson syndrome, which cluster in exons 2–9 and 25–28. Missense variants in a region spanning from exon 10 to 24, which encode the LEa-LF-LEb domains in the laminin β_2_ short arm, were found in 9 patients (64.3%) with isolated nephropathy (cases 4–13), despite the mutations in that region not being reported in previously reported classical Pierson syndrome patients with ocular or neurological manifestations (Supplemental Table 2). Furthermore, none of these 9 patients progressed to ESRD in the infantile period. Therefore, we hypothesized that variants in this region may be associated with the mild phenotype caused by *LAMB2* abnormalities.

## Secretion of laminin β_2_ short arm carrying p.R469Q, p.G699R, and p.R1078C in vitro.

**Figure 2:**
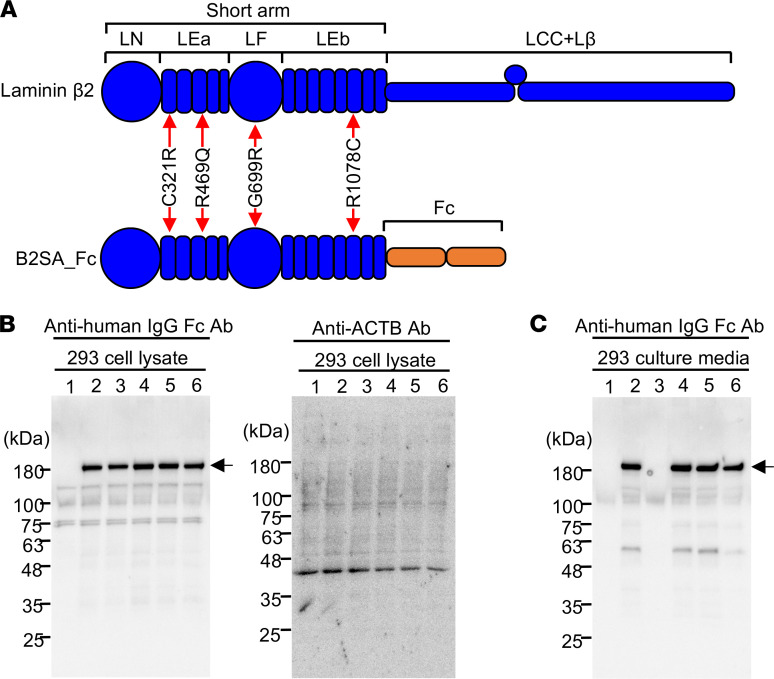
Secretion of laminin β_2_ variants in vitro. (**A**) Schematic diagrams of laminin β_2_ domains. Two-way arrows indicate positions of the missense variants in LEa, LF, and LEb domains. The β_2_ short arm carrying the variants was fused with an Fc-tag (B2SA_Fc). (**B**) Immunoblot analysis of WT and mutant laminin β_2_ short arm/Fc fusion proteins in lysates of HEK293 cells (left panel). Cell lysates of negative control (lane 1), B2SA_Fc (WT, lane 2), B2SA_C321R_Fc (p.C321R, lane 3), B2SA_R469Q_Fc (p.R469Q, lane 4), B2SA_G699R_Fc (p.G699R, lane 5), and B2SA_R1078C_Fc (p.R1078C, lane 6) were analyzed by immunoblot under reducing conditions. The recombinant proteins were detected using anti-human IgG Fc antibody as described in Methods. β-Actin (ACTB) was used as internal control (right panel). The variant proteins were expressed in the transfectants similar to WT. (**C**) Immunoblot analysis of WT and variant proteins in conditioned media of HEK293 cells. The variant proteins carrying R469Q, G699R, and R1078C were secreted with efficiency similar to WT.

To examine the impact of missense variants in the LEa-LF-LEb domains of the laminin β_2_ short arm, we searched for associated functional alterations. We chose 3 variants found in our patients, p.R469Q, p.G699R, and p.R1078C, affecting the LEa, LF, and LEb domains, respectively (Figure 1A). Although Arg469 and Gly699 are highly conserved among species, Arg1078 is replaced with His in the rhesus monkey and mouse (Figure 1B). Our previous study showed that some laminin β_2_ mutations result in impaired secretion (28). Therefore, we investigated whether the 3 β_2_ mutations influenced the secretion of laminin β_2_ by expressing N-terminal fragments of rat β_2_ mutants in HEK293 cells using a system in which the β_2_ short arm was fused to a human immunoglobulin Fc domain (Figure 2A). As shown in our previous study, although the p.C321R mutant was synthesized in the transfectants, its secretion was impaired (Figure 2, B and C). In contrast, the p.R469Q, p.G699R, and p.R1078C mutant fusion proteins were secreted into the medium similarly to the WT fusion protein. These results indicate that these 3 variants did not inhibit LM521 secretion.

## Heparin binding properties of mutant laminin β_2_ short arms.

**Figure 3:**
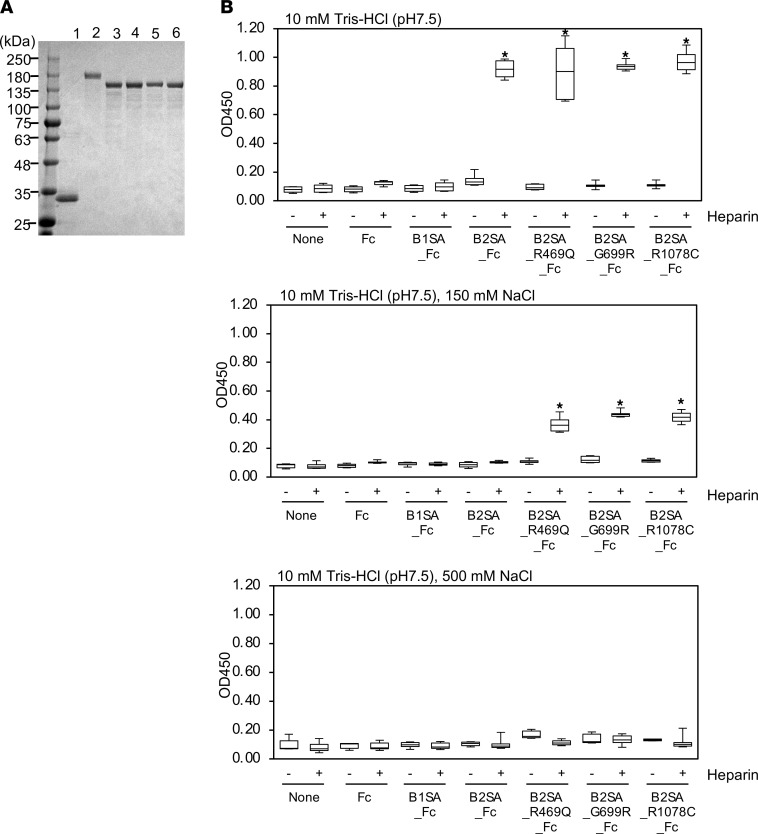
Solid phase binding assays of heparin to immobilized mutant laminin β_2_ short arm. (**A**) Variant laminin β_2_ short arm/Fc fusion proteins. Fc (lane 1), B1SA_Fc (β_1_ short arm, lane 2), B2SA_Fc (WT β_2_ short arm, lane 3), B2SA_R469Q_Fc (p.R469Q, lane 4), B2SA_G699R_Fc (p.G699R, lane 5), and B2SA_R1078C_Fc (p.R1078C, lane 6) purified from conditioned media of the transfectants were subjected to SDS-PAGE on a 5%–15% gel under reducing conditions. (**B**) Heparin binding of mutant laminin β_2_ protein in 10 mM Tris-HCl (pH 7.5) with 0 mM NaCl (upper panel), 150 mM NaCl (middle panel), and 500 mM NaCl (lower panel). Ninety-six-well microtiter plates were coated with 10 μg/mL of recombinant proteins. After blocking, the wells were incubated with 10 μg/mL of biotinylated heparin for 1 hour at room temperature. The recombinant Fc protein was used as control. The bound heparin was detected with horseradish peroxidase–conjugated streptavidin. Box-and-whisker plots show median, 25th and 75th percentiles, and minimum and maximum values (*n* = 6 from 3 independent experiments). Data were analyzed by 1-way ANOVA with Tukey’s multiple-comparison test, *P < 0.001.

*Lamb2*-knockout mice exhibit mislocalized anionic sites in the GBM, indicating that laminin β_2_ is associated with negative charge either directly or indirectly (29). The negative charge of the GBM is mainly imparted by heparan sulfate chains of the proteoglycan agrin (30, 31). Based on the known locations of both the laminin binding site and the glycosaminoglycan (GAG) attachment sites on agrin, together with super-resolution imaging data (32), the laminin β_2_ short arm could be capable of interacting with agrin’s negatively charged GAG side chains. Therefore, we investigated whether the laminin β_2_ variants bound to heparan sulfate using recombinant proteins. We purified recombinant rat laminin β_2_ short arms carrying the variants fused with human IgG_1_ Fc from conditioned media (Figure 3A). Human IgG_1_ Fc and mouse laminin β_1_ short arm fused with human IgG_1_ Fc were prepared as control proteins. SDS-PAGE analysis of the purified proteins revealed a single protein band corresponding to the expected size. Heparin, a highly sulfated form of heparan sulfate, was used for solid phase binding assays. Heparin acts as a cation exchanger because of its polyanionic features and can bind to cationic proteins in an ion strength–dependent manner. Whereas both WT and mutant laminin β_2_ short arms bound to heparin at low ionic strength, only the mutant forms bound under physiologic ionic conditions (Figure 3B). These results indicated that the variants increased the binding of laminin β_2_ to heparin under physiologic conditions. Our results also show that laminin β_1_ did not bind heparin, indicating that heparin binding is a characteristic feature of the β_2_ chain.

**Figure 4:**
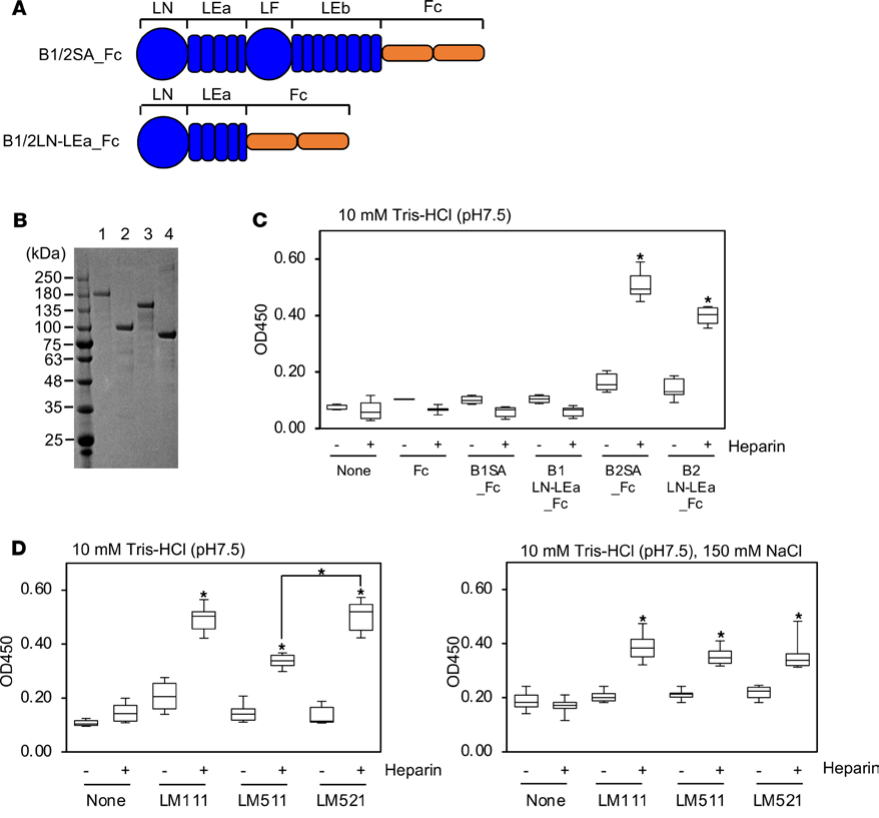
Characterization of heparin binding to the laminin β_2_ short arm. (**A**) Schematic diagrams of truncated laminin β_1_ and β_2_ short arms fused with an Fc-tag. (**B**) SDS-PAGE analysis of B1SA_Fc (β_1_ short arm, lane 1), B1LN-LEa_Fc (lane 2), B2SA_Fc (β_2_ short arm, lane 3), and B2LN-LEa _Fc (lane 4). (**C**) Heparin binding of β chain LN-LEa domains in 10 mM Tris-HCl (pH 7.5). Ninety-six-well microtiter plates were coated with 10 μg/mL of recombinant proteins. After blocking, 10 μg/mL of biotinylated heparin was applied to the wells and incubated for 1 hour at room temperature. The bound heparin was detected as described in Methods. Box-and-whisker plots show median, 25th and 75th percentiles, and minimum and maximum values (*n* = 6 from 3 independent experiments). Data were analyzed by 1-way ANOVA with Tukey’s multiple-comparison test, *P < 0.001. (**D**) The binding of heparin to whole laminin containing β_2_ chain in 10 mM Tris-HCl (pH 7.5), without (left panel) or with 150 mM (right panel) NaCl. Ninety-six-well microtiter plates were coated with laminin-111 (LM111), laminin-511 (LM511), and laminin-521 (LM521) at 10 μg/mL. Data were analyzed by 1-way ANOVA with Tukey’s multiple-comparison test, *P < 0.001 and are shown as box-and-whisker plots (*n* = 6 from 3 independent experiments).

To narrow the heparin binding region on the laminin β_2_ short arm, we produced truncated recombinant fragments (Figure 4, A and B). Solid phase binding assays were performed under low ionic conditions. Heparin bound to laminin β_2_ LN-LEa domains, indicating the heparin binding region is localized in the N-terminus (Figure 4C). These results suggest that the variants structurally influence the heparin binding affinity of the N-terminus under physiologic conditions rather than providing additional heparin binding sites. We next examined the heparin binding of trimeric laminins and found that under low ionic conditions, LM521 containing the β_2_ chain bound heparin significantly stronger than did laminin-511 containing the β_1_ chain (Figure 4D). There was no difference in heparin binding under physiologic conditions. These results indicate that laminin β_2_ influences heparin-binding properties of the whole laminin trimer.

**Figure 5:**
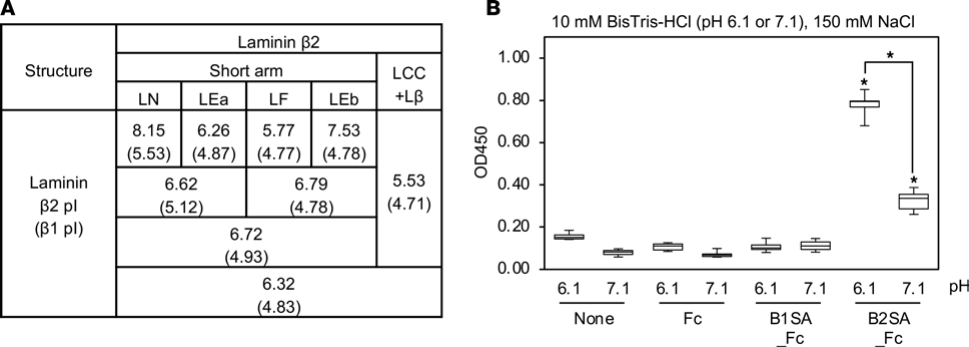
Characterization of laminin β_2_ chain. (**A**) Summary of isoelectric points (pI) of each domain of laminin β_1_ and β_2_ chains. (**B**) The extent of binding of heparin to laminin β_1_ and β_2_ short arms in 150 mM NaCl with 10 mM BisTris-HCl at pH = 6.1 or pH = 7.1, as indicated, was determined. Ninety-six-well microtiter plates were coated with the recombinant proteins at 10 μg/mL. After blocking, 10 μg/mL of biotinylated heparin was applied into the wells and incubated for 1 hour at room temperature. The bound heparin was detected as described in Methods. Box-and-whisker plots show median, 25th and 75th percentiles, and minimum and maximum values (*n* = 6 from 3 independent experiments). Data were analyzed by 1-way ANOVA with Tukey’s multiple-comparison test, *P < 0.001.

Although heparin bound to laminin β_2_ at low ionic strength, it is unlikely that there are low-salt conditions in vivo. Another factor that can modulate the charge of laminin β_2_ for heparin binding is the concentration of hydrogen ion (pH). To explore this, we first calculated the theoretical isoelectric point (pI) of each domain based on amino acid sequence (Figure 5A and Supplemental Figure 3). Of the laminin β_2_ domains, the pI of the LN domain was more than 7.4, which is the physiologic pH in blood. On the other hand, each pI of laminin β_1_ domains exhibited acidity. These results suggest that the laminin β_2_ LN domain has more positive charge under acidic conditions. Therefore, solid phase binding assays were performed under different pH conditions (Figure 5B). Heparin binding to the laminin β_2_ short arm was enhanced at pH = 6.1 versus pH = 7.1. These results indicate that laminin β_2_ modulates GBM charge in a pH-dependent manner.

## The binding of laminin β_2_ variants to laminin-111.

**Figure 6:**
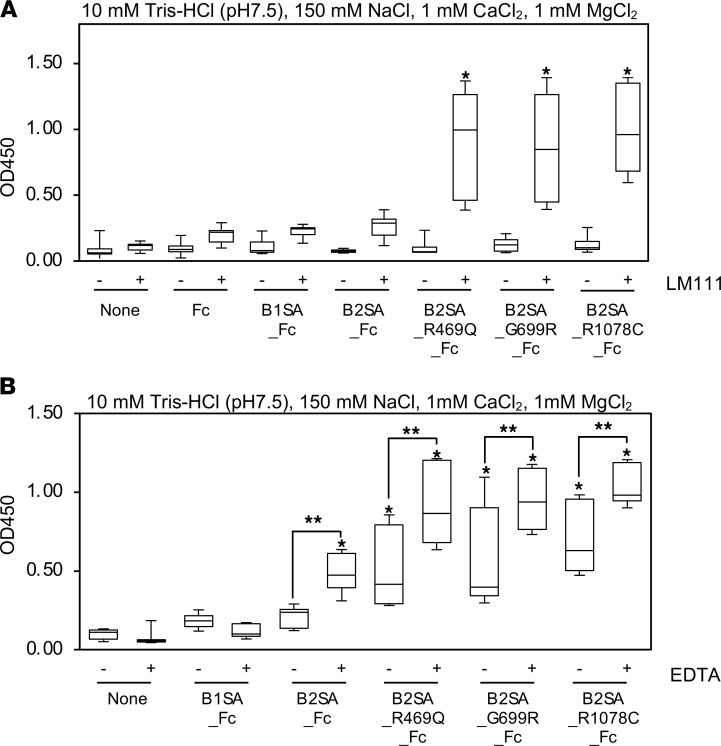
Solid phase binding assays of mutant β_2_ short arm to immobilized laminin-111. (**A**) The binding of mutant laminin β_2_ short arm/Fc fusion proteins in 10 mM Tris-HCl (pH = 7.5), 150 mM NaCl, 1 mM CaCl_2_, and 1 mM MgCl_2_. Ninety-six-well microtiter plates were coated with 10 μg/mL of EHS laminin-111. After blocking, the wells were incubated with 10 μg/mL of recombinant proteins for 1 hour at room temperature. The bound recombinant proteins were detected with anti-human IgG Fc antibody. Box-and-whisker plots show median, 25th and 75th percentiles, and minimum and maximum values (*n* = 12 from 4 independent experiments). Data were analyzed by 1-way ANOVA with Tukey’s multiple-comparison test, *P < 0.001. (**B**) Inhibitory effect of EDTA on the binding of mutant laminin β_2_ short arm/Fc fusion proteins to immobilized EHS laminin-111. The recombinant proteins were incubated in the presence of 10 mM EDTA. Data are shown as box-and-whisker plots (*n* = 9 from 3 independent experiments) with analysis by 1-way ANOVA with Tukey’s multiple-comparison test, *P < 0.001, **P < 0.05.

Laminins polymerize to form a network in BMs. The polymerization of laminins is mediated by the 3 short arms of the cross-shaped heterotrimer (33). The short arms are capped with LN domains that mediate the interactions among laminin trimers. To investigate the interaction of mutant laminin β_2_ short arms with other laminins, solid phase binding assays were performed using laminin-111 (Figure 6A). Laminin β_2_ mutants readily bound to laminin-111, but the WT β_2_ short arm did not bind at this concentration (64 nM). The binding of β_1_ and β_2_ LN domains to laminin-111 was also observed at higher concentration (128 nM), indicating that they bound less robustly than did the mutants (Supplemental Figure 4). Divalent cations such as Ca^2+^ are required for self-polymerization of laminins (33). To investigate the manner of laminin-111 binding, we also performed solid phase binding assays in the presence of EDTA (Figure 6B). Chelation of divalent cations significantly enhanced laminin-111 binding rather than inhibiting it. That the laminin β_2_ short arm bound to laminin-111 in a divalent cation-independent manner suggests that the variants unmask a novel laminin binding site.

## Adhesion of HEK293 cells to the laminin β_2_ short arm in vitro.

**Figure 7:**
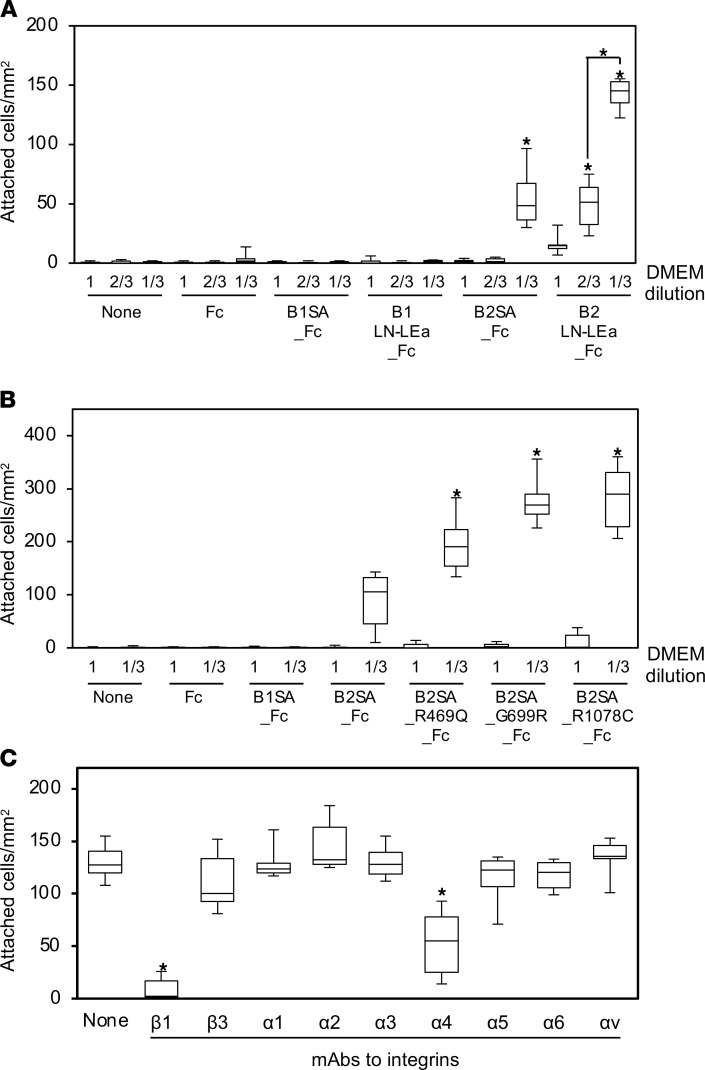
Analysis of cell adhesion to laminin β_2_. (**A**) Adhesion of HEK293 cells to the WT β_2_ short arm in diluted DMEM. Ninety-six-well microtiter plates were coated with 10 μg/mL of Fc (control tag protein), B1SA_Fc (β_1_ short arm), B1LN-LEa_Fc (β_1_ LN-LEa domains), B2SA_Fc (β_2_ short arm), and B2LN-LEa_Fc (β_2_ LN-LEa domains). After blocking, HEK293 cells were suspended in DMEM diluted with H_2_O and incubated in the wells at 37°C for 30 minutes. Attached cells were stained with Diff-Quik and counted. Box-and-whisker plots show median, 25th and 75th percentiles, and minimum and maximum values (*n* = 6 from 3 independent experiments). Data were analyzed by 1-way ANOVA with Tukey’s multiple-comparison test, *P < 0.001. (**B**) Adhesion of HEK293 cells to the mutant β_2_ short arms in the 1:3 DMEM dilution. Ninety-six-well microtiter plates were coated with 20 μg/mL of the mutant recombinant proteins. The number of attached cells is shown as box-and-whisker plots (*n* = 6 from 3 independent experiments) with analysis by 1-way ANOVA with Tukey’s multiple-comparison test, *P < 0.001. (**C**) Inhibitory effects of integrin-blocking antibodies on adhesion of HEK293 cells to β_2_ LN-LEa domains. HEK293 cells preincubated with function-blocking antibodies against the indicated integrin subunits were added to B2LN-LEa_Fc–coated wells. After incubation for 30 minutes, the attached cells were stained and counted. The attached cells were quantified and analyzed as described above. Data are shown as box-and-whisker plots (*n* = 6 from 3 independent experiments) with analysis by 1-way ANOVA with Tukey’s multiple-comparison test, *P < 0.001.

The heparin binding activity of laminin β_2_ led us to hypothesize that it binds to cell surface receptors containing heparan sulfate such as syndecans and glypicans. We therefore explored the cell adhesion activity of the laminin β_2_ short arm. HEK293 cells suspended in serum-free DMEM were plated on wells coated with the recombinant proteins. The cells weakly adhered to the laminin β_2_ LN-LEa domains but not to the full β_2_ short arm (Figure 7A). Based on the heparin binding at low ionic strength, the cell adhesion assay was next performed in diluted DMEM. The low ionic strength markedly promoted cell adhesion not only to the laminin β_2_ LN-LEa domains but also to the β_2_ short arm. The cells readily adhered to the laminin β_2_ LN-LEa domains, indicating that the cell attachment site is localized in the N-terminus. The laminin β_2_ mutants were more cell adhesive than the WT β_2_ short arm (Figure 7B). Thus, similar to heparin binding, the variants appeared to structurally influence cell-adhesive activity of the laminin β_2_ N-terminus. Furthermore, to clarify the mechanism of cell adhesion, we identified the receptors’ binding to the laminin β_2_ N-terminus. Although cell surface receptors carrying heparan sulfate were initially expected, the cell adhesion assays revealed divalent cation dependency, indicating the involvement of integrins. Cell adhesion assays were performed in the presence of function-blocking antibodies to integrins (Figure 7C). Anti–integrin β_1_ and α_4_ monoclonal antibodies significantly inhibited the adhesion of HEK293 cells to laminin β_2_ LN-LEa domains, indicating that the cell adhesion is mediated by integrin α_4_β_1_.

## Discussion

Recently, variants in *LAMB2* have been found in patients with nephropathy but without apparent extrarenal manifestations. These cases also present with less severe renal phenotypes. Proteinuria is often found in childhood, and some cases maintain renal function in adolescence, which is a clear contrast to the clinical course of typical Pierson syndrome. Electron microscopic analysis of kidneys of these patients demonstrated a thin or stratified GBM forming a basket weave pattern (Supplemental Figure 1 and ref. 13). Notably, laminin β_2_ in the GBM from a proteinuric patient carrying compound heterozygous truncating (p.Y76TfsTer) and missense (p.G699R) variants was detected (13), indicating that p.G699R did not cause a secretion defect. We noted that the distribution of missense variants causing Pierson syndrome and those of isolated nephropathy were different: variants in a region spanning from exon 10 to exon 24, which correspond to the LEa-LF-LEb domains in the laminin β_2_ short arm, were exclusively found in patients with isolated nephropathy. Although it is known that LN domains mediate the self-polymerization of laminins, the functions of the LEa-LF-LEb domains have been unclear. Here we found that the p.R469Q, p.G699R, and p.R1078C variants affecting the LEa-LF-LEb domains found in isolated nephropathy cases did not abrogate laminin secretion but altered heparin and laminin-111 binding. These data suggest that the LEa-LF-LEb domains regulate matrix interactions, and conformational changes in this segment lead to altered GBM structure. Because these variants do not cause extrarenal defects, the altered LEa-LF-LEb domains must maintain those laminin β_2_ functions required in ocular and neurological tissues. The present results suggest a novel mechanism underlying isolated nephropathy caused by *LAMB2* defects, which is clinically and genetically distinct from Pierson syndrome.

The glomerular filtration barrier is permeable to water and small molecules and selectively restricts macromolecules such as albumin (34). Because proteinuria precedes podocyte abnormalities in *Lamb2*-knockout mice, the GBM plays a critical role in selective filtration (29). The GBM had been believed to exhibit not only size selectivity but also charge selectivity to produce urine (31). However, because the reduction of negative charge using knockout mice does not affect the filtration barrier (35, 36), the hypothesis of charge selectivity has been challenged (37). Therefore, the mechanism whereby laminin β_2_ influences the size selectivity of the GBM is an important concept. In this study, WT laminin β_2_ bound heparin under not only low ionic but also acidic conditions, indicating that heparin binding is pH dependent. The pH dependency of laminin β_2_ binding to heparin is likely due to the basic pI of the β_2_ LN domain. The basic or neutral pI of the β_2_ LN domain is conserved in the mouse and human (Supplemental Figure 4) whereas that of the β_1_ LN domain is acidic. This may be why laminin β_1_ imperfectly compensates for the functions of β_2_, despite their structural similarity (38). Our solid phase binding assays also showed that the laminin β_2_ variants, but not WT β_2_, bound to heparin under neutral conditions. The recombinant proteins carrying p.R469Q, p.G699R, and p.R1078C bound similarly to heparin. The binding of mutant amino acid residues to heparin was minimal under physiologic conditions.

**Figure 8:**
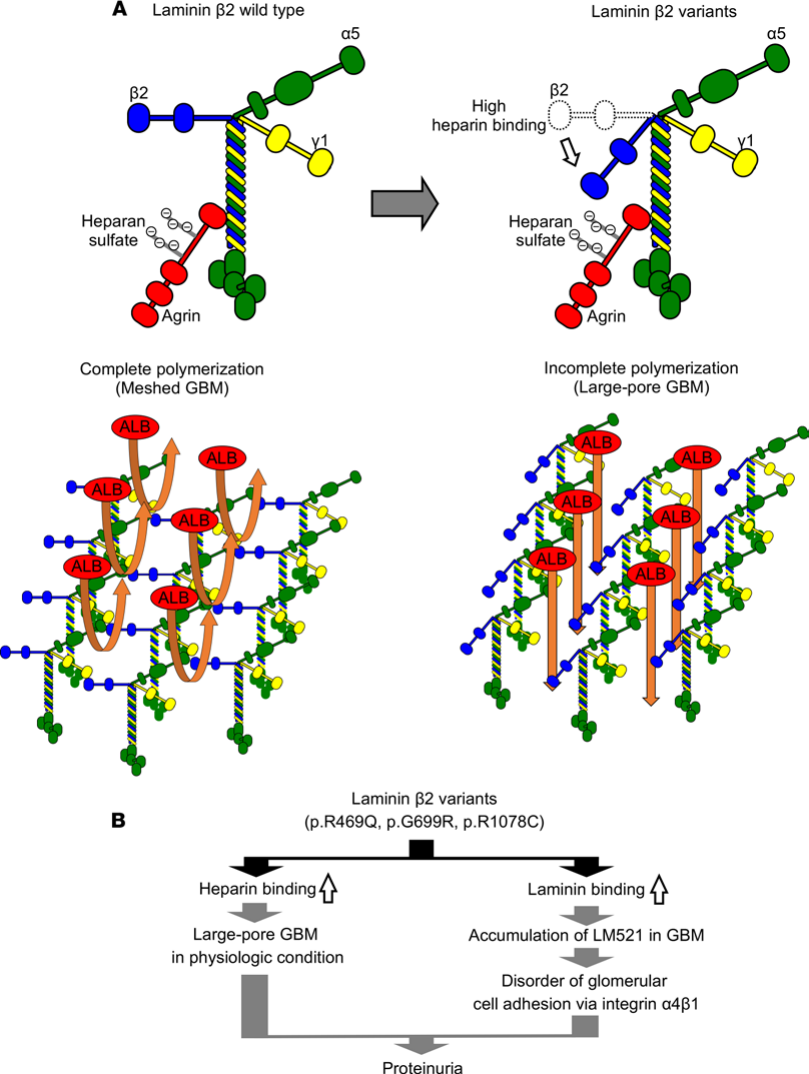
Schematic of laminin β_2_ activity in the GBM. (**A**) The disorder of permselectivity on GBM with β_2_ variants. The polymerization of laminin-521 containing WT β_2_ chain forms the GBM that restricts permeability of macromolecules such as albumin (ALB) (left). The N-terminus of β_2_ variants binds to heparan sulfates on agrin, when the LEa-LF-LEb variants are present (right). This binding disturbs laminin polymerization, resulting in larger GBM pores with increased permeability to albumin. (**B**) Dysfunction of GBM with laminin β_2_ variants. The heparin-binding activity of laminin β_2_ variants leads to a large-pore GBM under neutral conditions, resulting in proteinuria. Increased laminin binding also results in the accumulation of β_2_ variants in the GBM. The increased cell adhesive activity would affect glomerular cells.

The solid phase binding assay using truncated recombinant proteins showed that heparin binding sites are localized in the N-terminus of β_2_. The variants may lead to a conformational change in the β_2_ short arm, causing heparin binding sites to become exposed. The heparin binding property of laminin β_2_ allows us to hypothesize a model in which the short arm of β_2_ variants binds to heparan sulfate on agrin in the GBM (Figure 8A). This binding seems to disturb laminin polymerization under physiologic conditions, resulting in a large-pore GBM that can be permeable to macromolecules such as albumin. Similar to the β_2_ variants, the WT β_2_ short arm binds to heparan sulfate under acidic conditions. To test this model, future investigations on the effect of pH variation in mice would be required.

Once laminins are secreted into the extracellular space, they polymerize to form a meshwork via interactions among LN domains of the α, β, and γ short arms (33). The pairings of α-α, α-β, α-γ, and β-γ are possible in the presence of Ca^2+^ (39). Although the LN domain of β_2_ binds to the N-termini of α_1_, _2_, _5_; β_1_, _2_; and γ_1_, _3_, our solid phase binding assays showed that WT β_2_ short arm weakly bound to laminin-111. On the other hand, the mutant β_2_ short arms readily bound to laminin-111 in a calcium-independent manner. These in vitro results suggest a model in which the variants expose a cryptic laminin binding site in the β_2_ short arm under physiologic conditions. Structural or biochemical analyses using samples from patients or knock-in mice carrying these variants are required to confirm the model.

Our results indicate that laminin β_2_ serves as a potentially novel cell-adhesive ligand for integrin α_4_β_1_. Integrin α_4_β_1_ plays a major role in the regulation of immune cell recruitment to inflamed endothelia and other sites of inflammation. Previous studies have reported that integrin α_4_β_1_ is a receptor for vascular cell adhesion molecule-1 (VCAM-1) and fibronectin (40). In fibronectin, integrin α_4_β_1_ recognizes a motif containing the sequence EILDVPST within the alternatively spliced connecting segment-1 (41), with the LDV sequence being most critical (42). Homology search of the rat laminin β_2_ amino acid sequence revealed that 2 LDVs are localized in the LN and LCC domains, respectively. The N-terminal LDV sequence is likely exposed to integrin α_4_β_1_ because the position is close to the signal peptide cleavage site. However, this LDV is conserved in rodents but not humans. We also searched for the LDV sequence in human laminin β_2_ and found that it is localized in the LF domain. This LDV is conserved in mammals except for rodents. Pulido et al. reported the crystal structure of the laminin β_2_ LF domain (43). Because the position of the LDV is located at the molecular surface, integrin α_4_β_1_ would be able to bind to the sequence. Our recent study showed that the β_2_ short arms are polymerized toward the center of the GBM, suggesting that it is difficult for the binding site to be accessible to integrins (32). The excessive deposition of β_2_ variants in the GBM may expose adhesive sites for integrin α_4_β_1_–positive cells (Figure 8B). In this regard, expression of integrin α_4_β_1_ was detected in the endothelial cells and mesangial cells of glomeruli (Supplemental Figure 5). Therefore, the exposure of the integrin binding site might strengthen cell adhesion, leading to glomerular dysfunction. Integrin α_4_β_1_ is a major cell surface receptor on immune cells such as lymphocytes. Moreover, Jurkat and Raji cells derived from human lymphocytes adhere to laminin β_2_ via integrin α_4_β_1_ (data not shown). The nephritogenic immune responses are driven by CD4^+^ T cells (44). CD4^+^ T cells spontaneously adhere within uninflamed glomerular capillaries (45), and the accumulation of immune cells such as CD4^+^ T cells, neutrophils, and macrophages is often observed in inflamed glomeruli (44). In addition to VCAM-1 on glomerular endothelial cells, laminin β_2_ may also serve as an adhesive ligand for inflammatory cells in nephritis.

Increased laminin deposition in the GBM has been reported in a *Col4a3^–/–^* mouse model of Alport syndrome (46, 47). LAMB1 and LAMA2 are normally found in the mesangial matrix, but ectopic deposition is detected in Alport GBM (46). Furthermore, increased laminin α_5_ mRNA was detected in Alport glomeruli (47). The increased deposition of laminin was also reported in patients with Alport syndrome (48). These increases may be due to increased production or altered turnover of extracellular matrix molecules triggered by instability caused by the collagen abnormality. We hypothesize that the mechanism of GBM laminin anomalies observed in Alport syndrome is different from that predicted here due to variations in the LEa-LF-LEb domains of LAMB2.

The variants in the LEa-LF-LEb domains found in isolated nephropathy are not common, but the allele frequencies of some variants are around 1% in specific ethnic populations. For example, the frequency of c.2095G>C (p.G699R; rs28364667) is 0.012 in the East Asian population and 0.007 in Japanese databases. Variant c.3071C>T (p.P1024L; rs368506627) is observed at 0.0098 in the South Asian population (gnomAD, ref. 20). Many Mendelian disorders, such as Pierson syndrome, are caused by highly penetrant rare variants. Recent empirical evidence has shown that variants at low frequency or rare variants are associated with complex diseases. The recessive nature of these variants and the residual function of the mutant proteins make it difficult to determine their pathogenicity. Mouse models with altered LEa-LF-LEb domains to mimic the defect or exome- or whole genome–based variant analysis in large cohorts, including several target populations, will be required to further uncover the contribution of disease-related variants in this region to the pathogenesis of proteinuric kidney disease.

## Methods

### Antibodies and regents.

Monoclonal antibodies against human integrin α_2_ (P1E6), α_3_ (P1B5), α_4_ (P4G9), α_5_ (P1D6), α_6_ (P5G10), and α_V_ (P3G8) (Developmental Studies Hybridoma Bank) were purified on Protein G Sepharose (Cytiva) from conditioned media of hybridoma cells purchased from Developmental Studies Hybridoma Bank. Anti–integrin α_1_ (FB12), β_1_ (6S6), and β_3_ (25E11) antibodies were purchased from Merck. Anti–β-actin antibody conjugated with peroxidase was from Medical & Biological Laboratories Co. Ltd. Alexa Fluor 647–conjugated rat monoclonal antibody against mouse integrin α_4_ (R1-2) and isotype control rat IgG2bκ (RTK4530) were purchased from BioLegend. Anti-podocin polyclonal antibody (catalog ab50339) was from Abcam. Human recombinant laminin-111 (LM111), laminin-511 (LM511), and laminin-521 (LM521) were purchased from BioLamina. Mouse EHS laminin was from BD Biosciences and Takako Sasaki (Oita University School of Medicine, Oita, Japan). Biotinylated heparin with an average mass of 12.5 kDa was from Celsus Laboratories, Inc.

### Construction of expression vectors.

cDNA clones encoding full-length mouse laminin β_1_ (provided by Albert E. Chung, University of Pittsburgh, Pittsburgh, Pennsylvania) and rat laminin β_2_ (generated in-house) were used to construct expression vectors. The fragments encoding laminin β_1_ LN-LEa domains (B1N) and short arm (B1SA) were amplified using primer sets described in Supplemental Table 3 and subcloned into a human IgG_1_ Fc expression vector. The EcoRI-XbaI fragment encoding the laminin β_2_ LN-LEa domains (B2N) was subcloned into a human IgG_1_ Fc expression vector. To construct the β_2_ short arm expression vector, the fragment encoding laminin β_2_ LF-LEb domains was amplified using primer sets described in Supplemental Table 3 and subcloned into the XbaI site of the B2N_Fc expression vector. The R469Q, G699R, and R1078C human laminin β_2_ variants were separately engineered into analogous sites of the rat cDNA by site directed mutant PCR using primer sets described in Supplemental Table 3. Laminin β_2_ carrying the C321R variant was prepared in our previous study (28).

### Preparation of serum-free conditioned media and cell lysates.

HEK293 cells were obtained from the American Type Culture Collection. The cells were maintained in DMEM containing 10% fetal calf serum. To express WT and mutant proteins, the expression vectors were transiently transfected into the cells in 24-well plates using Lipofectamine 2000 (Thermo Fisher Scientific). The next day, the growth media were replaced with 500 μL of serum-free medium. After 3 days’ culture, the conditioned media were harvested and clarified by centrifugation twice at 100*g* for 5 minutes and 9730*g* for 10 minutes. Then 20 μL of conditioned media were applied for immunoblotting. The transfectants were also lysed with 500 μL of 10 mM Tris-HCl (pH 7.5), 150 mM NaCl, 10 mM EDTA, 1% NP-40, and protease inhibitor cocktail (Merck). The cell lysates were clarified by centrifugation once at 9730*g* for 10 minutes. Then 20 μL of cell lysate was applied for immunoblotting.

### Immunoblotting.

Cell lysates and conditioned media were separated on 5%–20% gradient gels (ATTO) under reducing conditions and transferred to a PVDF membrane (Merck). Conditioned media and lysates were immunoblotted with anti-human IgG_1_ Fc antibody (Jackson ImmunoResearch catalog 109-066-098). The bound antibodies were visualized with the ECL Western blotting detection system (Cytiva). The images of bands were captured with WSE-6100 Lumino Graph I (ATTO).

### Expression and purification of recombinant proteins.

HEK293 cells transfected with the expression vectors were selected using 100 μg/mL Zeocin (Thermo Fisher Scientific). All further cell culture was carried out in the presence of the antibiotic. The selected cells were grown to confluence in culture dishes with DMEM containing 10% FBS. The confluent cells were incubated in serum-free DMEM for 4 days. The conditioned media were harvested and clarified by centrifugation twice at 100*g* for 5 minutes and 9730*g* for 10 minutes. The recombinant proteins were purified from the conditioned culture media by Protein A Sepharose (Cytiva). The eluted fractions were pooled and dialyzed against PBS(-). The purified proteins were separated by SDS-PAGE using 5%–20% gels under reducing conditions. The separated proteins were stained with Coomassie Brilliant Blue (nacalai tesque).

### Solid phase binding assays.

Heparin binding assays were carried out with recombinant proteins coated onto high-protein-binding capacity 96-well microtiter plates (IWAKI). The plates were blocked with PBS(-) containing 1% BSA for 1 hour at room temperature. A total of 10 μg/mL of biotinylated heparin in 10 mM Tris-HCl (pH 7.5) containing 0, 150, and 500 mM NaCl or 10 mM BisTris-HCl (pH 6.1 and 7.1) containing 150 mM NaCl was added into the wells. After incubation for 1 hour at room temperature, the bound heparin was detected by addition of streptavidin-conjugated horseradish peroxidase, followed by addition of 1 mg/mL *o*-phenylenediamine and 0.012% H_2_O_2_. The absorbance was measured at 450 nm with Multiskan GO microplate spectrophotometer (Thermo Fisher Scientific). For laminin-111 binding assay, 96-well microtiter plates were coated with 10 μg/mL of EHS laminin-111. After blocking with PBS(-) containing 1% BSA, 10 μg/mL of Fc-tagged recombinant proteins in the binding buffer (10 mM Tris-HCl pH 7.5, 150 mM NaCl, 1 mM CaCl_2_, 1 mM MgCl_2_ containing 0.05% Tween 20) were incubated at room temperature for 1 hour. The bound recombinant proteins were detected with a biotinylated anti-human IgG Fc antibody (Jackson ImmunoResearch) and quantified as described above.

### Cell attachment assays.

For adhesion assays, 96-well microtiter plates (IWAKI) were coated with recombinant proteins and blocked with PBS(-) containing 1% BSA. The dissociated HEK293 cells were suspended in serum-free DMEM and plated at 2 × 10^4^ cells/50 μL/well. After incubation at 37°C for 0.5 or 1 hour, the attached cells were stained with Diff-Quik (SYSMEX). The stained cells were counted using image cytometer BZ-X800 (Keyence). To identify the receptors for laminin β_2_, 10 μg/mL of monoclonal antibodies against different integrins were preincubated individually with cells in a volume of 50 μL of serum-free medium (2 × 10^4^ cells/well) at room temperature for 10 minutes. The preincubated cells were transferred to plates coated with laminin β_2_ LN-LEa domains fused with Fc-tag (B2N_Fc) and then incubated further at 37°C for 1 hour. After incubation, the attached cells were quantified as described above.

### Staining of renal biopsy specimens.

Kidney biopsy specimens were fixed in 10% neutral buffered formalin and embedded in paraffin. The tissues were sectioned and stained with periodic acid–methenamine silver stain–Masson’s trichrome and periodic acid–Schiff.

### Electron microscopy.

After fixation in glutaraldehyde fixative (1.6% v/v paraformaldehyde, 2.5% v/v glutaraldehyde, 0.2 M sodium cacodylate buffer pH 7.4, 5 mM CaCl_2_, 10 mM MgCl_2_), tissues were embedded in epon resin and sectioned. Imaging was performed using a HITACHI H-7100.

### Immunohistochemistry.

C57BL/6J mice (72 weeks old, male) were purchased from Charles River Laboratories Japan. Mice were sacrificed under deep anesthesia with Thiopental. Mouse kidneys were frozen in Tissue-Tek optimum cutting temperature compound (Sakura Finetek) and were cut at 7 μm in a cryostat. After blocking with 10% normal goat serum, the sections were incubated with rat monoclonal antibody against mouse integrin α_4_ and rabbit polyclonal antibody against podocin. The integrin α_4_ antibody conjugated with Alexa Fluor 647 was used. Rabbit IgG was detected with secondary antibody conjugated with Alexa Fluor 594 (Thermo Fisher Scientific catalog A11037). After several washes, sections were mounted in 90% glycerol containing 0.1× PBS and 1 mg/mL *p*-phenylenediamine. Images were captured using a BZ-X800 (Keyence).

### Statistics.

Comparisons between groups were analyzed using 1-way ANOVA with Tukey’s multiple-comparison test. Statistical analyses were performed in R Studio (Version 1.3.1093). Significance, set at *P* < 0.05, is depicted as follows in figures: *P < 0.001, **P < 0.05.

### Study approval.

Informed consent was obtained from all the members of a family according to the guidelines of the Ethics Committee of Yamagata University (protocol number 2018-190) and the University of New Mexico School of Medicine. Clinical data of patients, including laboratory data, imaging data, and kidney biopsy, were obtained for diagnostic purposes and with informed consent. Animal studies were approved by the Animal Research Committee of Tokyo University of Pharmacy and Life Sciences (P20-33).

## Author contributions

TH, YH, and YK were responsible for the overall design of the study. KT, SU, HM, MM, YY, KH, MN, M Hisano, YA, and KM performed the experiments and analyzed the data. JHM prepared cDNA clone encoding full-length rat laminin β_2_. TH, YH, M Hattori, HL, JHM, and YK reviewed the results and wrote the manuscript. All authors offered constructive comments.

## Supplementary Material

Supplemental data

## Figures and Tables

**Table 1:**
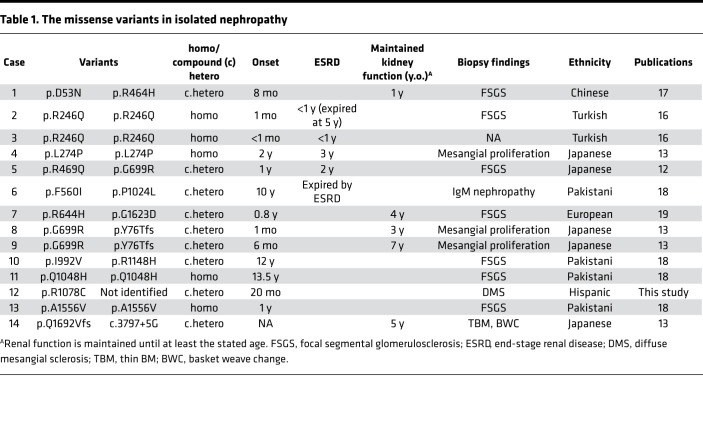
The missense variants in isolated nephropathy

## Version 1 03/22/2021

Electronic publication
