# Supplementary material for: Laminin β2 variants associated with isolated nephropathy that impact matrix regulation
Source: JCI Insight. 2021 Mar 22;6(6):e145908. doi: 10.1172/jci.insight.145908 (PMC8026196; doi:10.1172/jci.insight.145908)
Supplement: Supplemental data [file jciinsight-6-145908-s063.pdf]

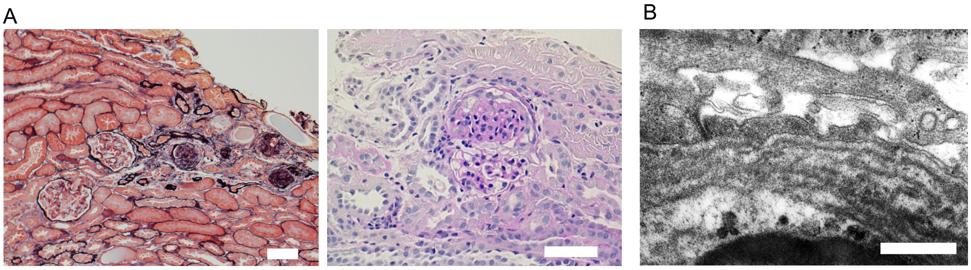

Figure S1. Glomerulus with laminin β2 carrying p.R469Q and p.G699R variants. (A) PAM-Masson trichrome (left panel) and PAS (right panel) staining of the patient renal biopsy sample. Bar: 100 µm. (B) Ultrastructural analysis of the patient GBM. Bar: 200 nm.

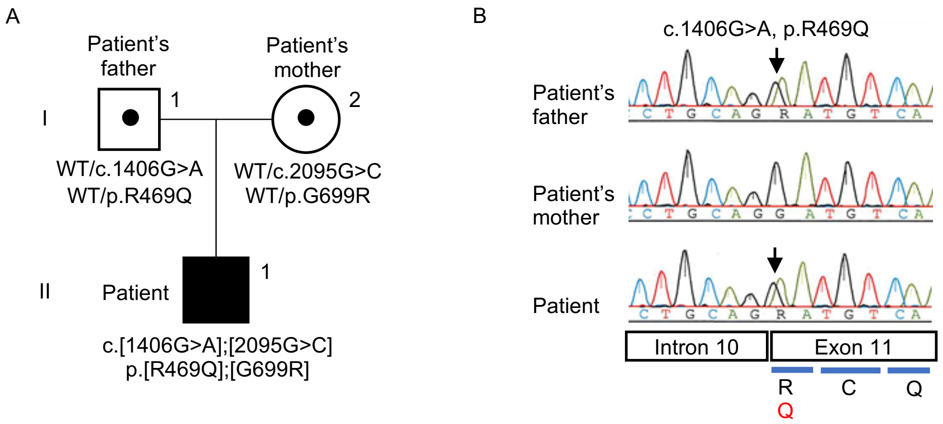

C

NNSplice (www.fruitfly.org/seq_tools/splice.html)

	Score	Intron	Exon
WT	0.97	gatggttctccttccctgcag	gatgtcaatgtaatgcacgg
c.1406G>A	0.96	gatggttctccttccctgcag	aatgtcaatgtaatgcacgg

Alternative Splice Site Predictor (ASSP) (http://wangcomputing.com/assp/)

					Activations		
	Position (bp)	Sequence	Score	Intron GC	Alt./Cryptic	Constitutive	Confidence
WT	Constitutive acceptor	ttccctgcagGATGTCAATG	12.890	0.529	0.247	0.741	0.666
c.1406G>A	Constitutive acceptor	ttccctgcagAATGTCAATG	11.839	0.529	0.362	0.627	0.423

Figure S2. Characterization of c.1406G>A in *LAMB2* gene of a FSGS patient. (A) Family pedigree of the FSGS patient. (B) Sanger sequence chromatograms of patient family members. The region cording exon 11 of *LAMB2* gene were amplified from the genomic DNA using polymerase chain reaction. The sequence reactions were analyzed on an ABI PRISM 3100 Genetic Analyzer (PE Applied Biosystems, Foster City, CA) with the BigDye Terminator Cycle Sequencing Ready Reaction Kit (PE Applied Biosystems). Arrows indicate the mutated sites in these chromatograms. (C) Splicing prediction score of acceptor sites between intron 10 and exon 11 calculated by NNSplice and Alternative Splice Site Predictor (ASSP).

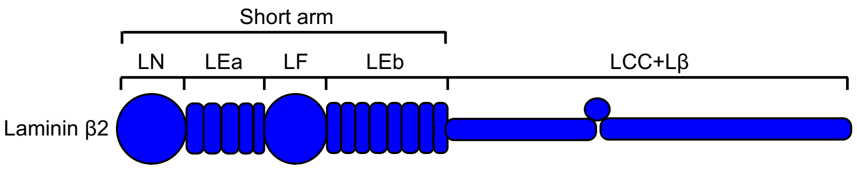

<table>
<tr><td>Species</td><td colspan="5">Human</td></tr>
<tr><td rowspan="3">Structure</td><td colspan="5">Laminin β2</td></tr>
<tr><td colspan="4">Short arm</td><td rowspan="2">LCC+Lβ</td></tr>
<tr><td>LN</td><td>LEa</td><td>LF</td><td>LEb</td></tr>
<tr><td rowspan="4">Lamb2 pI (Lamb1 pI)</td><td>7.20 (5.53)</td><td>6.65 (4.92)</td><td>5.03 (4.74)</td><td>7.08 (4.67)</td><td rowspan="3">5.23 (4.73)</td></tr>
<tr><td colspan="2">6.81 (5.15)</td><td colspan="2">6.30 (4.67)</td></tr>
<tr><td colspan="4">6.50 (4.90)</td></tr>
<tr><td colspan="5">6.07 (4.82)</td></tr>
</table>

<table>
<tr><td>Species</td><td colspan="5">Mouse</td></tr>
<tr><td rowspan="3">Structure</td><td colspan="5">Laminin β2</td></tr>
<tr><td colspan="4">Short arm</td><td rowspan="2">LCC+Lβ</td></tr>
<tr><td>LN</td><td>LEa</td><td>LF</td><td>LEb</td></tr>
<tr><td rowspan="4">Lamb2 pI (Lamb1 pI)</td><td>7.61 (5.53)</td><td>6.40 (4.87)</td><td>5.56 (4.77)</td><td>7.18 (4.78)</td><td rowspan="3">5.53 (4.71)</td></tr>
<tr><td colspan="2">6.66 (5.12)</td><td colspan="2">6.54 (4.78)</td></tr>
<tr><td colspan="4">6.66 (4.93)</td></tr>
<tr><td colspan="5">6.25 (5.53)</td></tr>
</table>

<table>
<tr><td>Species</td><td colspan="5">Rat</td></tr>
<tr><td rowspan="3">Structure</td><td colspan="5">Laminin β2</td></tr>
<tr><td colspan="4">Short arm</td><td rowspan="2">LCC+Lβ</td></tr>
<tr><td>LN</td><td>LEa</td><td>LF</td><td>LEb</td></tr>
<tr><td rowspan="4">Lamb2 pI (Lamb1 pI)</td><td>8.15 (5.53)</td><td>6.26 (4.87)</td><td>5.77 (4.94)</td><td>7.53 (4.85)</td><td rowspan="3">5.53 (4.72)</td></tr>
<tr><td colspan="2">6.62 (5.12)</td><td colspan="2">6.79 (4.88)</td></tr>
<tr><td colspan="4">6.72 (4.99)</td></tr>
<tr><td colspan="5">6.32 (4.86)</td></tr>
</table>

Figure S3. Summary of isoelectric point (pI) on domains of laminin β1 and β2 chains in different species.

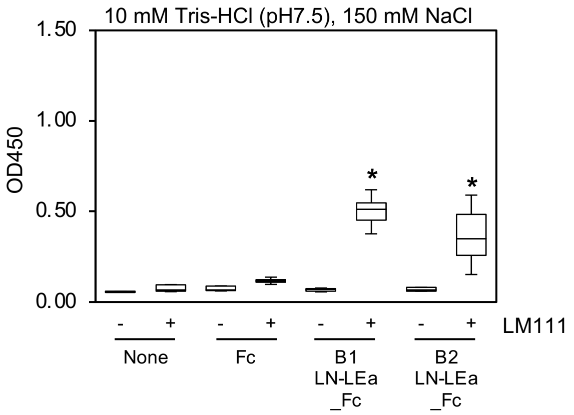

Figure S4. The binding of laminin β LN-LEa domains/Fc fusion proteins to immobilized laminin-111 in 10 mM Tris-HCl (pH7.5), 150 mM NaCl, 1mM $CaCl_2$, 1mM $MgCl_2$. The recombinant Fc protein was used as control. Ninety six-well microtiter plates were coated with 10 μg/ml of EHS laminin-111. After blocking, the wells were incubated with 10 μg/ml of recombinant proteins for 1 hour at room temperature. The bound recombinant proteins were detected as described in Methods. Box-and-whisker plots show median, 25th and 75th percentiles, and minimum and maximum values (n = 6 from 3 independent experiments). Data were analyzed by one-way ANOVA with Tukey's multiple-comparison test, $*P < 0.001$.

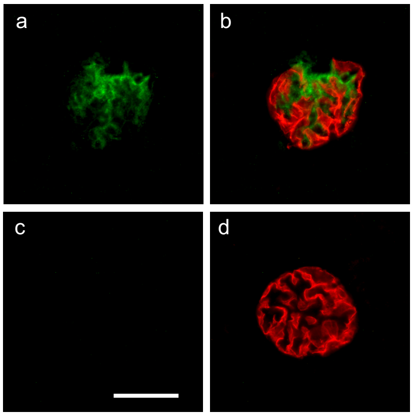

Figure S5. Expression of integrin α4 in mouse glomerulus. Frozen tissue sections of kidney were prepared from 72 weeks aged mice. They were stained with Alexa Fluor 647-conjugated antibody to integrin α4 (a and b: green). The sections were simultaneously stained with antibody to Podocin to label podocytes (b, d: red). Merged images of integrin α4 and Podocin staining are shown in b and d panels. Rat $IgG_{2b\varkappa}$ conjugated with Alexa Fluor 647 is used as control (c and d: green). The signals of Alexa Fluor 647 were converted to green in the figure. Integrin α4 is expressed in glomerular endothelial cells of aged kidney, suggesting that laminin β2 influences to the cells via integrin α4β1. Bar: 50 μm.

Figure S5

Table S1. Frequencies of c.2095G>C (p.(G699R)) in *LAMB2* gene				
Population	Allele count	Allele number	Number of homozygotes	Allele Frequency
European (non-Finish)	2	129162	0	0.00001548
European (Finish)	0	25122	0	0
Latino	1	35436	0	0.0002613
Ashkenazi Jewish	0	10370	0	0
African	0	24964	0	0
South Asian	8	30616	0	0.0002613
East Asian	234	19954	1	0.01173
Other	3	7224	0	0.0004153
total	248	282848	1	0.0008768

Table S2. The missense mutations in classical Pierson syndrome			
Exon/Intron	DNA variant	Predicted amino acid	Publications
Missense or in-frame deletion mutations			
Exon2	c.235-237delGTC	p.V79del	(1)
Exon2	c.240T>G	p.S80R	(2, 3)
Exon4	c.416T>C	p.L139P	(2)
Exon4	c.440A>G	p.H147R	(4)
Exon4	c.447-449delTAT	p.I149del	(5)
Exon5	c.482T>C	p.L161P	(6)
Exon5	c.499G>T	p.D167Y	(5, 7)
Exon5	c.536C>T	p.S179F	(8, 9)
Exon7	c.736C＞T	p.R246W	(2, 5, 10-13)
Exon7	c.737G>A	p.R246Q	(10, 14, 15)
Exon8	c.961T>C	p.C321R	(14)
Exon8	c.970T>C	p.C324R	(16)
Exon9	c. 1129T>C	p.C377R	(17)
Exon9	c.1144G>C	p.A382P	(10)
Exon25	c.3982G>C	p.G1328R	(15)
Exon26	c.4177C>T	p.L1393F	(14)
Exon28	c.4616G>A	p.R1539Q	(6, 18)
Truncating mutations			
Exon11	c,1477delT	p.C493fs*	(10)
Exon13	c.1723C>T	p.R575*	(5)
Exon14	c.1888C>T	p.Q630*	(17)
Exon17	c.2283_2286delCTCT	p.S762fs*	(8)
Exon24	c.3450_3451 ins A	p.D1151fs*	(9)
Exon29	c.4904_4905delCA	p.T1635fs*	(18)
Exon29	c.4907_4908delAG	p.E1636fs*	(6)
Exon31	c.5182C>T	p.Q1728*	(1)
Exon31	c.5197C>T	p.Q1733*	(2)

The previously reported missense *LAMB2* mutations found in patients presenting apparent extrarenal (ocular or neuromuscular) manifestation(s).

REFERENCES

1. Matejas V, et al. A syndrome comprising childhood-onset glomerular kidney disease and ocular abnormalities with progressive loss of vision is caused by mutated *LAMB2*. *Nephrol Dial Transplant*. 2006;21(11):3283-6.
2. Matejas V, et al. Mutations in the human laminin beta2 (*LAMB2*) gene and the associated phenotypic spectrum. *Hum Mutat*. 2010;31(9):992-1002.
3. Lehnhardt A, et al. Pierson syndrome in an adolescent girl with nephrotic range proteinuria but a normal GFR. *Pediatr Nephrol*. 2012;27(5):865-8.
4. Mohney BG, et al. A novel mutation of *LAMB2* in a multigenerational mennonite family reveals a new phenotypic variant of Pierson syndrome. *Ophthalmology*. 2011;118(6):1137-44.
5. Bredrup C, et al. Ophthalmological aspects of Pierson syndrome. *Am J Ophthalmol*. 2008;146(4):602-11.
6. Minamikawa S, et al. Molecular mechanisms determining severity in patients with Pierson syndrome. *J Hum Genet*. 2020;65(4):355-62.
7. Kagan M, et al. A milder variant of Pierson syndrome. *Pediatr Nephrol*. 2008;23(2):323-7.
8. Choi HJ, et al. Variable phenotype of Pierson syndrome. *Pediatr Nephrol*. 2008;23(6):995-1000.
9. Mbarek IB, et al. Novel mutations in steroid-resistant nephrotic syndrome diagnosed in Tunisian children. *Pediatr Nephrol*. 2011;26(2):241-9.
10. Sadowski CE, et al. A single-gene cause in 29.5% of cases of steroid-resistant nephrotic syndrome. *J Am Soc Nephrol*. 2015;26(6):1279-89.
11. Zenker M, et al. Human laminin beta2 deficiency causes congenital nephrosis with mesangial sclerosis and distinct eye abnormalities. *Hum Mol Genet*. 2004;13(21):2625-32.
12. Machuca E, et al. Genotype-phenotype correlations in non-Finnish congenital nephrotic syndrome. *J Am Soc Nephrol*. 2010;21(7):1209-17.
13. Schapiro D, et al. Panel sequencing distinguishes monogenic forms of nephritis from nephrosis in children. *Nephrol Dial Transplant*. 2019;34(3):474-85.
14. Hasselbacher K, et al. Recessive missense mutations in *LAMB2* expand the clinical spectrum of *LAMB2*-associated disorders. *Kidney Int*. 2006;70(6):1008-12.
15. Tahoun M, et al. Mutations in *LAMB2* Are Associated With Albuminuria and Optic Nerve Hypoplasia With Hypopituitarism. *J Clin Endocrinol Metab*. 2020;105(3).
16. Falix FA, et al. A novel mutation of laminin beta2 (*LAMB2*) in two siblings with renal failure. *Eur J Pediatr*. 2017;176(4):515-9.
17. Zhang H, et al. *LAMB2* mutation with different phenotypes in China. *Clin Nephrol*. 2017;87 (2017)(1):33-8.
18. Nagano C, et al. Comprehensive genetic diagnosis of Japanese patients with severe proteinuria. *Sci Rep*. 2020;10(1):270.

Table S3. Primer sets

Protein	Primer	Sequence (5'-3')
B1N	MLB1LEa02	CGTCTAGAGTGGTGAAGTAGTAACC
	MLB1LN01	CGGGATCCGCCACCATGGAAGGGCCCCTCTCCTCT
B1SA	B1LEb_01	CTAGGATCCATTGGGGCTTTCCCAGCTGCCAGC
	B1LEb_04	GACTCCTAGGCCGGGTGTGCAGTCAGGAAAGACCCC
B2SA	RB2IV01	GGAATTCCGAACAAGTACAGCCTGGCTAC
	RB2LEb_06	GACTTCTAGACCGGGGTGACAAGCAGGAAAAACACC
B2SA_R469Q	RB2LN_01	TATCCAGCCACCGGTGACCTGTTG
	RB2LEa_02	TGTGCCCCTTGAGTTACACTGACACTGCTGACATCCTCGAGGGTTGCT
	RB2LEa_01	AGCAACCCTCGAGGATGTCAGCAGTGTCAGTGTAACTCAAGGGGCACA
	RB2LEb_08	ACCAAAGGCACCGGTTCGGCAGAG
B2SA_G699R	RB2LF_02	TGGGGTTTCAGGATGGGCACGTCCCCGTGTTCCAGTCAATTTCAACTTCAG
	RB2LF_01	CTGAAGTTGAAATTGACTGGAACACGGGGACGTGCCCATCCTGAAACCCCA
B2SA_R1078C	RB2LEb_04	GTTCCAAAAGTTGGGGGCACAACAGTCGCAACTGAGGCCTTGGAC
	RB2LEb_01	GTCCAAGGCCTCAGTTGCGACTGTTGTGCCCCCAACTTTTGGAAC
